# Flowers a Remedy

**Published:** 1875-02

**Authors:** 


### FLOWERS A REMEDY.

A beautiful bunch of bright flowers was one day brought to a dying lady; her face brightened up in a moment and a beautiful smile lighted up her countenance as she expressed her gratitude toward the friend, who herself was an invalid, for her kind remembrance. Let beautiful flowers be everywhere, on the mantel of the sick, in the cell of the prisoner, on the tea table, the breakfast table, the dinner table—the very sight of them enlivens and elevates and purifies. Those who have growing flowers should pinch off every leaf the moment it begins to wilt or fade, and cut off every flower before it begins to go to seed, this prevents the rapid exhaustion of the strength of the stem and freshens all the other buds and adds largely to the crop.

